# Gallstone Dislodgement in the Airway during ERCP: A Case Report and Review of the Literature

**DOI:** 10.1155/2020/1519243

**Published:** 2020-08-20

**Authors:** Hisham Hurreiz, Amira Babikir, Edward Black

**Affiliations:** ^1^Al Ain Hospital, Al Ain, UAE; ^2^Tawam Hospital, Al Ain, UAE

## Abstract

Endoscopic retrograde cholangiopancreatography (ERCP) is a diagnostic and therapeutic procedure with many studied complications. We are presenting a rare complication of ERCP in choledocholithiasis: gallstone dislodging into the airway upon retrieval. The patient is a 37-year-old female admitted with obstructive jaundice. She was evaluated, and her management plan included a referral for an ERCP to extract the impacted common bile duct stones. Upon retrieval of the gallstone, it fell out the basket and lodged into the airway which was confirmed on bronchoscopy and successfully retrieved. This report describes successful management of a rare but potentially dangerous complication of ERCP to remove impacted CBD stones. The possible complications of delayed removal or inability to remove gallstones from the airway have yet to be studied and reported but are likely to include recurrent chest infections, bronchiectasis, and empyema of the lung.

## 1. Introduction

Endoscopic retrograde cholangiopancreatography (ERCP) is a diagnostic and therapeutic procedure with many studied complications. Some of which include pancreatitis, bleeding, perforation, and cholangitis. It is commonly used to evaluate the biliary system for dilatation as well as locate tumors and impacted gallstones in obstructive jaundice patients. It is also used to obtain biopsies for tissue diagnosis as well as administer therapeutic measures such as placement of biliary stents and extraction of impacted common bile duct stones. We are presenting a rare complication of ERCP in choledocholithiasis: gallstone dislodging into the airway upon retrieval.

## 2. Case History

**Figure 1:**
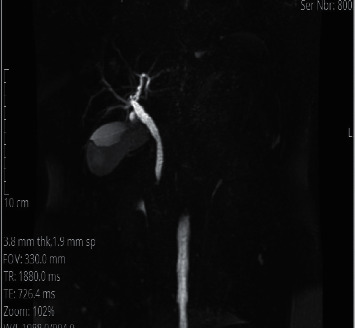
MRCP, coronal view, showing the dilated CBD with a distal filling defect.

**Figure 2:**
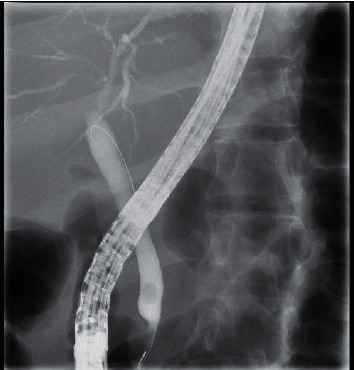
Fluoroscopy during ERCP showing the dilated CBD and contrast reaching the hepatic ducts.

A 37-year-old female, known to have cholelithiasis, presented to our emergency department with recurrent attacks of upper abdominal pain associated with nausea. On examination, her abdomen was soft with localised tenderness in the right upper quadrant. Her blood investigations showed hyperbilirubineamia. She underwent ultrasound (US) examination and magnetic resonance cholangiopancreatography (MRCP) (Figure 1) which revealed dilatation of the biliary tree with stones seen within the common bile duct (CBD). She was therefore referred for an ERCP to remove the stone impacted in the distal CBD. She underwent ERCP (Figure 2) 4 days after admission. The initial cholangiogram confirmed the presence of a stone in the distal CBD. A papillotomy was carried out with balloon sweeping and extraction of the CBD stone via endoscopic dormia basket under monitored anaesthesia using propofol for sedation. During extraction, the stone dislodged from the basket and was aspirated into the upper airway. A rigid bronchoscopy was performed, and this demonstrated the presence of the stone in the trachea which was successfully extracted. The patient recovered well after the procedure and underwent laparoscopic cholecystectomy within the same admission. She was discharged home 2 days later, and on subsequent review at the surgical clinic 2 weeks after the surgery, she demonstrated an uneventful recovery.

## 3. Discussion

The most frequent complications of endoscopic retrograde cholangiopancreatography (ERCP) and endoscopic biliary sphincterotomy are pancreatitis (3.5%), cholangitis (1%), haemorrhage (1.3%), and duodenal perforation (0.6%) [1–4]. A number of less common adverse events have also been described including cardiopulmonary complications, contrast allergy, impaction of a retrieval basket, and numerous other events reported in only small numbers of patients or individual case reports. These unusual adverse events, which may be difficult to manage, can be associated with significant morbidity and mortality [5, 6]. Factors affecting morbidity and mortality during ERCP include operator experience and patient comorbidity [7].

No previous reports exist of gallstone dislodgement into the airway during ERCP. There were, however, reports of possible sequelae after the presence of gallstone in chest cavity rather than within the bronchopulmonary tree, namely, biliothoraces and cholecystothoracic fistulas [8].

Another report documented complications of basket malfunction or disruption in which metallic fragments of the basket were left in situ and removed at a later date using endoscopy [9]. The risk of leaving gallstones within the bronchial tree are theoretically similar to presence of other foreign bodies, which includes pneumonia, atelectasis, bronchiectasis, respiratory distress if the stone is big enough to obstruct the upper airway, as well as bleeding or erosion into nearby structures, but to our knowledge, there are no reports in the literature describing the sequelae of the presence of gallstones in the airway and demonstrating if any difference exists between the different types of stones.

## 4. Conclusion

This report describes successful management of a rare but potentially dangerous complication of ERCP to remove impacted CBD stones. In the presence of experienced endoscopy and bronchoscopy teams, such stones can be safely removed endoscopically, avoiding the risk of major surgery. The possible complications of delayed removal or inability to remove gallstones from the airway have yet to be studied and reported but are likely to include recurrent chest infections, bronchiectasis, and empyema of the lung.
